# Epigenetics: possible applications in climate-smart crop breeding

**DOI:** 10.1093/jxb/eraa188

**Published:** 2020-04-11

**Authors:** Serena Varotto, Eleni Tani, Eleni Abraham, Tamar Krugman, Aliki Kapazoglou, Rainer Melzer, Aleksandra Radanović, Dragana Miladinović

**Affiliations:** 1 Department of Agronomy, Food, Natural Resources, Animals, and the Environment, University of Padova, Agripolis, Viale dell’Università, Padova, Italy; 2 Department of Crop Science, Laboratory of Plant Breeding and Biometry, Agricultural University of Athens, Athens, Greece; 3 Laboratory of Range Science, School of Agriculture, Forestry and Natural Environment, Aristotle University of Thessaloniki, Thessaloniki, Greece; 4 Institute of Evolution, University of Haifa, Haifa, Israel; 5 Institute of Olive Tree, Subtropical Crops and Viticulture (IOSV), Department of Vitis, Hellenic Agricultural Organization-Demeter (HAO-Demeter), Lykovrysi, Greece; 6 School of Biology and Environmental Science and Earth Institute, University College Dublin, Belfield, Dublin, Ireland; 7 Institute of Field and Vegetable Crops, Maksima Gorkog, Serbia; 8 University Clermont Auvergne, France

**Keywords:** Abiotic stress, breeding, chromatin, climate-smart, crops, DNA methylation, epigenetic changes, small RNA

## Abstract

To better adapt transiently or lastingly to stimuli from the surrounding environment, the chromatin states in plant cells vary to allow the cells to fine-tune their transcriptional profiles. Modifications of chromatin states involve a wide range of post-transcriptional histone modifications, histone variants, DNA methylation, and activity of non-coding RNAs, which can epigenetically determine specific transcriptional outputs. Recent advances in the area of ‘-omics’ of major crops have facilitated identification of epigenetic marks and their effect on plant response to environmental stresses. As most epigenetic mechanisms are known from studies in model plants, we summarize in this review recent epigenetic studies that may be important for improvement of crop adaptation and resilience to environmental changes, ultimately leading to the generation of stable climate-smart crops. This has paved the way for exploitation of epigenetic variation in crop breeding.

## Teaser

Intense breeding has narrowed crop genetic diversity. Epigenetic diversity could emerge as new source of phenotypic variations to improve crop adaptation to a changing environment and generate stable climate-smart crops.

## Introduction

Plant response to abiotic stresses is complex and involves multiple mechanisms, activated and controlled by massive changes in gene expression and nuclear organization (Budak *et al.*, 2015). Evidence from model plants indicates that epigenetic modifications, some of them inherited by the next generation, play an important role in this response. However, the role of epigenetic variation in adaptation of crops to abiotic stress is still not well understood (Niederhuth and Schmitz, 2014).

Epigenetics is a fascinating field of genetics, completely meddling with classical knowledge of the interaction between the hereditary material (DNA/genes) and the phenotype, and puzzling scientists for decades. According to the definition of Arthur Riggs and his colleagues, epigenetics is ‘the study of mitotically and/or meiotically heritable changes in gene function that cannot be explained by changes in DNA sequence’ (Russo *et al*., 1996). Epigenetic changes, which involve DNA methylation, histone modifications, chromatin remodelling, and activity of small RNAs (sRNAs), are thus heritable, but do not follow the known patterns of inheritance (Bräutigam *et al.*, 2013). There is a certain advantage in examining epigenetics in plants as compared with animals. In plants there is no need to set up a dedicated germ line, because the germ line is developed from somatic cells (Feng *et al.*, 2010; Rival and Jaligot, 2012). Moreover, in plants, active maintenance mechanisms enable the transfer of epigenetic information throughout gametogenesis, then fertilization, and early embryogenesis (Rival and Jaligot, 2012). However, epigenetic states can often be unstable. Therefore, to achieve plant improvement that may persist over generations, we need to learn which epigenetic states can be stably transmitted (Kinoshita and Seki, 2014).

Abiotic stresses negatively impact plant growth and development, with severe effects on crop yield leading to huge economic losses. Since most epigenetics studies have been carried out in model plants, particularly Arabidopsis (Pecinka *et al.*, 2020), the accumulated knowledge on the role of epigenetic regulation in response to the environment has led to an increased interest in the role of epigenetics in crop resilience to abiotic stress. Here we summarize recent studies focusing on epigenetic mechanisms, particularly those involved in crop response to environmental changes, which could be important for crop improvement for better adaptation to environmental changes and breeding of climate-smart crops.

## Epigenetic mechanisms and marks

Chromatin conformation influences the accessibility of DNA sequences, such as coding and regulatory DNA sequences, to the transcriptional machinery thus intervening in regulation of gene expression (Li *et al.*, 2008). DNA accessibility for transcription is mediated by nucleosome positioning: each core nucleosome is made up of a histone octamer comprising two copies each of histone H2A, H2B, H3, and H4 and about 146 DNA base pairs in two turns. Unpackaged DNA between nucleosomes, with varying length depending on the nucleosomes’ compaction level, is associated to H1, the linker histone. Interaction of histones with DNA is also influenced by reversible covalent post-translational modifications of the histone tails protruding from the nucleosome core particle. The suite of post-translational modifications that dynamically regulate the level of chromatin condensation and DNA accessibility is known as the histone code (Wei *et al.*, 2017). Histone acetylation and deacetylation and histone methylation and demethylation are two well-characterized reversible plant histone modifications. Acetylation marks, such as histone 3 lysine 9 acetylation (H3K9ac), histone 3 lysine 14 acetylation (H3K14ac), and histone 3 lysine 36 acetylation (H3K36ac), are associated with gene activation. Histone deacetylation at the same residues is associated to transcriptional repression. Histone methylation can have a gene activating or repressing role depending on the site of modifications: histone H3 lysine 4 (H3K4) and H3K36 methylation are related to gene expression, while H3K9 and H3K27 methylation are related to gene repression and heterochromatin formation. In plants, both transposable elements and repetitive sequence-enriched heterochromatic regions are marked by histone H3 lysine 9 monomethylation and dimethylation (H3K9me1 and H3K9me2), which maintain the repressive transcriptional state. Heterochromatin regions are also associated with H3K27me1, while the trimethylated state of H3K27 (H3K27me3) exerts a repressive role in euchromatin regions. In particular, H3K27me3 is preferentially enriched in the entire transcribed region of inactive genes (Liu *et al.*, 2010).

Chromatin can be modified in plants at the level of DNA sequence by DNA methylation that occurs in CG, CHG, and CHH (H=A, T or C) contexts through distinct pathways. METHYLTRANSFERASE 1 (MET1) and CHROMOMETHYLASE 3 (CMT3) are plant enzymes responsible for the maintenance of CG and CHG methylation, respectively (Zhang *et al.*, 2018). CHH methylation is established *de novo* through two pathways. The RNA-dependent DNA methylation pathway involves biogenesis of small interfering RNAs (24-nt siRNAs) that are targeted to corresponding genomic loci by ARGONAUTE (AGO) family members, which in turn are methylated via DOMAINS REARRANGED METHYLTRANSFERASE2 (DRM2). A second pathway requires CHROMOMETHYLASE 2 (CMT2) through interaction with DECREASE IN DNA METHYLATION1 (DDM1) in histone H1-enriched chromatic regions (Zemach *et al.*, 2013). Additionally, DNA methylation can be actively removed by a family of bifunctional methyl-cytosine glycosylases-apurinic/apyrimidinic lyases, through a base excision repair mechanism (Penterman *et al.*, 2007). DNA methylation may affect gene expression, regulate imprinting, and activate transposable elements and transposable element-associated genes, particularly in response to environmental cues (Law and Jacobsen, 2010).

Our understanding of epigenetic determinants that underlie transcriptional outputs in model plants has been complemented by accumulating evidence of stress-induced changes that are regulated by sRNAs (Khraiwesh *et al.*, 2012; Popova *et al.*, 2013). Plant sRNAs are produced by different pathways, and different categories of short non-coding RNAs were demonstrated to contribute to *de novo* DNA methylation, gene silencing, and other epigenetic processes (Matzke and Mosher, 2014). In addition to sRNAs, long non-coding RNAs (lncRNAs) are transcripts longer than 200 nucleotides that can interact with both nucleic acids and proteins and act as scaffolds for the formation of specific functional complexes in the nucleus.

**Fig. 1.**
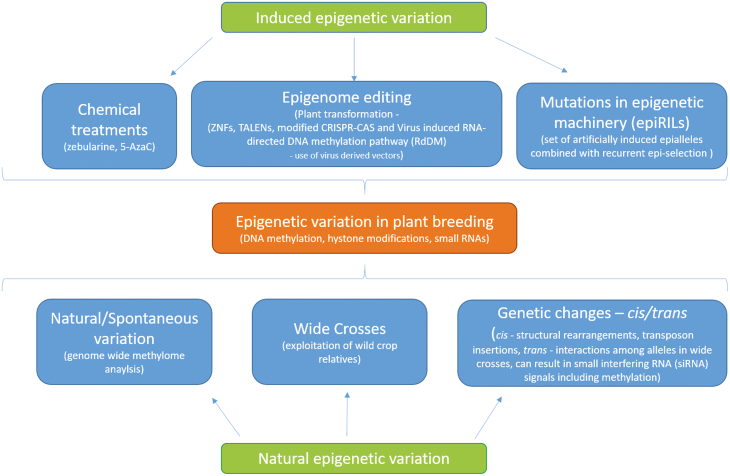
Epigenetic variations that could be used in plant breeding.

Taken together, a wide range of histone post-translational modifications, histone variants, DNA methylation, and activity of non-coding RNAs can alter chromatin configuration resulting in various chromatin states that epigenetically determine specific transcriptional outputs in plant cells. Chromatin states can vary in response to environmental stimuli, allowing the plant cells to fine tune their transcriptional profiles to better adapt transiently or lastingly to the surrounding environment (Fig. 1).

## The role of epigenetics in gene expression under abiotic stress

Plants are constantly exposed to environmental stresses, including low water and nutrient availability, extreme temperatures and light intensities, and soil properties such as salinity and heavy metal content. To cope with climate change and resulting increasing occurrences of unpredictable environmental conditions, plants have developed genetic and epigenetic mechanisms that enable them to withstand single or combined stresses and their interactions (Shanker and Venkateswarlu, 2011). Thus, understanding this complexity in crops requires knowledge of the genetic and epigenetic bases of responses to environmental changes. In one example of such research, Brzezinka *et al.* (2016) used priming by heat stress as a model to dissect the memory of environmental stresses in Arabidopsis in order to identify genes that are specifically required for heat stress memory, but not for the initial responses to heat. The authors identified the *FORGETTER1* (*FGT1*) gene, and found that the FGT1 protein binds directly to a specific class of heat-inducible genes and ensures that the heat-inducible genes are always accessible and active by modifying the way the DNA containing these genes is packaged. Their findings could lead to new approaches in crop breeding programmes for enhancing the resistance to abiotic stress, as knowledge of stability and heritability features of epigenetic marks and epigenetic regulatory mechanisms is crucial for breeding applications (Gallusci *et al.*, 2017).

The above-mentioned research has set a solid base for better understanding different mechanisms underlying variation influencing plant/crop productivity. Some examples related to crops will be further discussed in this review (Table 1).

**Table 1.** Epigenetic mechanisms involved in crop response to different abiotic stresses

Crop	Abiotic stress	Epigenetic mechanism(s)	Reference
Maize	Drought	Modifications of H3K4me3 and H3K9ac dynamics	Forestan *et al.* (2018)
		Enrichment in H3K36me3, H3K9ac, and H3K4me3	Xu *et al.* (2017)
	Heat	H3K4me2 and H3K9ac alterations	Hou *et al.* (2019)
		Increased histone acetylation and decreased H3K9me3	Wang *et al.* (2015b)
	Cold	Enrichment in H3K9ac and decrease in DNA methylation and H3K9me2	Hu *et al.* (2012)
		Reduction in histone acetylation in euchromatin-associated gene regions	Hu *et al.* (2011)
		DNA demethylation	Steward *et al.* (2002)
Wheat	Heat	Increased histone demethylation of the various genes	Wang *et al.* (2016)
	Salinity	Hypermethylation of cytosines at *HKT* genes	Kumar *et al.* (2017)
		5-mC depletion	Zhong *et al.* (2009)
Barley	Drought	Hc-siRNA-mediated hyper-methylation at CYTOKININ-OXIDASE 2.1 promoter	Surdonja *et al.* (2017)
		Increase in H3 and loss in H3K9me2	Temel *et al.* (2017)
		Accumulation of miR408 transcripts	Kantar *et al.* (2010)
Rice	Drought	Hypomethylation	Gayacharan and Joel (2013)
		Up-regulation of miR408 expression	Mutum *et al.* (2013)
		Site-specific DNA methylation	Wang et al. (2011*a*, *b*)
	Salinity	Demethylation at promoter region of *OsMYB91* gene and rapid histone modifications at *OsMYB9* locus	Zhu *et al.* (2015)
		DNA methylation	Karan *et al.* (2012), Ferreira *et al.* (2019
Soybean	Drought	miR1514a modulation of a NAC transcription factor transcript	Sosa-Valencia *et al.* (2017a)
		Up-regulation of isomiRNAs	Kulcheski *et al.* (2011)
	Heat	Hypomethylation of cytosine	Hossain *et al.* (2017)
Pea	Drought	Hypermethylation of cytosine residues	Labra *et al.* (2002)
Chickpea	Drought	Accumulation of miR408 transcripts	Hajyzadeh *et al.* (2015)
	Drought + Salinity	Accumulation of miRNAs at root apex	Khandal *et al.* (2017)
Cowpea	Drought	Increase of P5CS transcripts and very low expression of vun-miR5021 and vun-miR156b-3p	Shui *et al.* (2013)
Bean	Drought	Dicistronic arrangement of miR398a and miR2119	De la Rosa *et al.* (2019)
Faba bean	Drought	Increased DNA demethylation	Abid *et al.* (2017)
Alfalfa	Drought	Overexpression of miR156	Arshad *et al.* (2018)
Rapeseed	Heat	Increased DNA demethylation	Gao *et al.* (2014)
	Salinity	Increased DNA demethylation	Marconi *et al.* (2013)
Tomato	Drought	RNA-dependent DNA methylation	Benoit *et al.* (2019)
		Increased Asr1 and Asr2 expression due to demethylation of putative regulatory and transcribed regions	González *et al.* (2011, 2013)
	Cold	Increased DNA methylation	Zhang *et al.* (2016)

## Plant memory: adaptation to climatic change transmitted across generations

Creating climate-smart crops also requires knowledge of how epigenetic changes are transmitted across generations. It is widely accepted that an important part of these epigenetic modifications includes priming or memory, which is involved in improved capacity to withstand future stresses even if not primed by the same stress. However, since priming can affect plant growth and development, this phenomenon is not always observed. Thus, plants employ mechanisms to elucidate whether to memorize or to forget (Crisp *et al.*, 2016; Baier *et al.*, 2019; Mozgova *et al.*, 2019). In Arabidopsis, it has been shown that plant progeny preserve an adaptive epigenetic memory of temperature conditions of their ancestors (Whittle *et al.*, 2009). Zhong *et al.* (2013) showed clear associations between increase in ambient temperature, the plant epigenetic system, and siRNA biogenesis. The authors have found that moderate temperature increase had a transgenerational inhibitory effect on sense transgene-mediated post-transcriptional gene silencing/siRNA biogenesis. This warmth-induced epigenetic memory was maintained for at least three generations, with rapidly declining strength over further generations. Contrary to this, transgenerational epigenetic memory as a response to a pathogen or UV light showed a slower decrease in strength over several generations (Molinier *et al.*, 2006). Suter and Widmer (2013) showed that in Arabidopsis exposure to abiotic stress during several generations can induce heritable, potentially adaptive phenotypic changes, which were maternally and paternally inherited. Because the observed effects depended on plant genotype, the authors suggested an interaction between genetic background and inheritance of induced epigenetic patterns.

## Epigenetics responses to drought and temperature stresses in agronomically important crops

### Maize

Climate change and frequent occurrences of both biotic and abiotic stresses have become a constant threat for global production of maize (*Zea mays* L.), which is a principal cereal crop cultivated worldwide. It has recently been found that histone marks, in particular H3K4me3 and H3K27me3, may function as memory marks for stress-responsive genes and transcription factors in maize (Forestan *et al.*, 2018). Low water availability is an important environmental factor affecting maize yield. In order to evaluate how maize modulates its response to drought and recovery from drought stress, transcriptomic and genome-wide chromatin data were integrated (Forestan *et al.*, 2018). This study revealed the existence of multiple chromatin-mediated levels of gene transcriptional regulation in response to osmotic stress, involving non-coding RNAs (Lunardon *et al.*, 2016; Forestan *et al.*, 2016), as well as histone modifications of H3K4me3 and H3K9ac dynamics (Forestan *et al.*, 2018). Furthermore, genome-wide screening was conducted to identify lncRNA collections of drought stress-responsive maize transcripts expressed in different tissues (Zhang *et al.*, 2014). LncRNAs were then classified as either sRNA precursors or other non-coding RNAs through alignment with other sRNA databases. Xu *et al.* (2017) identified genes with expressed natural antisense transcripts (NATs), a complex class of regulatory RNAs, in two maize inbred lines that carry multiple loci responsible for drought tolerance and in two recombinant inbred lines generated from these two parental lines that are fixed for combinations of loci that confer either high or low drought tolerance. Although the function of NATs is not well understood in plants, Xu *et al.* (2017) found 1769 NAT pairs in two maize inbred lines, as well as in the two derivative recombinant inbred lines. Interestingly, Xu *et al.* (2017) also reported that NATs that correlate with stress response were significantly hypomethylated and included fewer transposable element sequences relative to non-NAT genes. In addition, at their genomic loci NATs appeared to be enriched in H3K36me3, H3K9ac, and H3K4me3, but not in H3K27me3, thus exhibiting an open chromatin configuration (Xu *et al.*, 2017).

Heat stress constrains maize growth and causes significant reduction in crop yield. At the chromatin level, heat stress could induce programmed cell death and modulate chromatin structure, increasing histone acetylation and decreasing H3K9me3 (Wang *et al.*, 2015*b*). Moreover, in maize seedlings, application of a short-term heat stress induces dynamic H3K4me2 and H3K9ac alterations in promoter regions, which were associated with heat stress factor (Hsf) and rRNA gene up-regulation, accompanied by perturbations of cell membranes and increase in reactive oxygen species (Hou *et al.*, 2019).

On the other hand, low temperature affects maize productivity especially if occurring in early stages of plant development. Hu *et al.* (2011) observed that treatment of maize with the histone deacetylase (HDAC) inhibitor trichostatin A under cold stress conditions strongly inhibits the induction of the maize cold-responsive genes *ZmDREB1* and *ZmCOR413*, while the up-regulation of the *ZmICE1* gene in response to cold stress was less affected by cold treatments. These results indicated that HDACs positively regulate the expression of the cold-induced *ZmDREB1* gene through histone modification and chromatin conformational changes and that this activation was both gene and site selective. In the genome of maize seedlings exposed to cold stress, both a global DNA demethylation and a reduction in histone acetylation in euchromatin-associated gene regions were observed (Steward *et al.*, 2002; Hu *et al.*, 2011). A detailed analysis at specific genomic repeats indicated that cold stress unsilenced selectively and transiently tandem repetitive sequences coupled with an enrichment in H3K9ac and decrease in DNA methylation and H3K9me2. In addition, nucleosome remodelling was observed at the same tandem repeat genomic regions (Hu *et al.*, 2012).

### Wheat

Wheat (*Triticum aestivum* L.) is one of the most important crops grown for human consumption. It exceeds any other cereal, including rice and maize, in total world growing area and total world food supply, with total growing area of 214.79 million ha in 2018 (FAO, 2018). It is grown in a wide range of ecogeographic regions around the globe, which requires acclimating its physiological responses to the pressures of abiotic and biotic stresses. Wheat yields are specifically affected when plants are exposed to stress at the reproductive stage (Begcy and Dresselhaus, 2018). Analysis of the wheat whole genome expression pattern (e.g. ‘expression atlas’) based on 850 RNA-seq samples derived from 32 tissues sampled at different growth stages and/or under different stress treatments revealed higher average methylation status in lowly expressed genes (Appels *et al.*, 2018). Furthermore, expression range patterns were correlated with the distribution of the repressive H3K27me3 (trimethylated histone H3 lysine 27) and with the active H3K36me3 and H3K9ac (acetylated H3K9) histone marks (Appels *et al.*, 2018). Gardiner *et al.* (2015) showed that methylation patterns in wheat are not equally distributed across the A, B, and D sub-genomes, reflecting patterns of methylation of progenitor species.

Several studies on epigenetic modifications occurring in wheat in response to abiotic stress were published in the past few years. Heterochromatic small interfering RNA (hc-siRNA) and microRNA (miRNA), small regulatory RNAs, have been shown to be involved in wheat drought stress response (Budak *et al.*, 2015). For example, 2055 putative targets were identified for 113 conserved durum miRNAs and 131 targets for four novel durum miRNAs that putatively contribute to genotypic stress tolerance (Liu *et al.*, 2017). As for salt stress, differential epigenetic modifications in specific genes such as *HKTs* (high-affinity potassium transporters) were found in shoots and roots of wheat genotypes differing in their sensitivity to this stress (Kumar *et al.*, 2017).

### Barley

Barley (*Hordeum vulgare* L.) is a major cereal grown in temperate climates globally, with total production of 141.75 million tons in 2018 (FAO, 2018). Despite the fact that barley inherently exhibits resilience to harsh climates, abiotic stress factors can inhibit the performance of the crop. Terminal drought stress during grain filling is the major abiotic factor that limits crop yield in barley. Stress-specific 24mer size hc-siRNA was found in the promoter regions of the barley cytokinin-oxidase 2.1 gene (*HvCKX2.1*) in the caryopsis exposed to terminal drought (Surdonja *et al.*, 2017). The authors found that under terminal drought the level of DNA methylation of this gene was increased. Interestingly, seeds derived from the drought-stressed mother plant had a fast germination rate. As also shown in wheat, when barley is exposed to drought and salt stress, numerous differently methylated sites were induced in leaves compared with roots (Chwialkowska *et al.*, 2016, Konate *et al.*, 2018). Hemi-methylations, representing single CHG or simultaneous CHG and CG asymmetric methylation, were also more abundant in leaves than in roots, whereas full methylations, indicating mainly symmetric CG methylation, were more frequent in roots (Chwialkowska *et al.*, 2016). A gene involved in *de novo* DNA methylation, *HvDRM*, was down-regulated in leaves and its expression was not altered in roots of plants exposed to drought stress (Chwialkowska *et al.*, 2016). The authors found that in barley DNA methylation level was higher than in other crops such as rapeseed, rice, and maize. This could be a consequence of a high content of repetitive elements in the barley genome (Mascher *et al.*, 2017). This phenomenon was also observed in angiosperms in which genome-wide DNA methylation levels were found to be related to the proliferation of repetitive elements (Niederhuth *et al.*, 2016). On a chromatin level, denser nucleosome packaging was observed in barley plants that were exposed to drought, and *HSP17* was identified as one of the drought-responsive genes (Temel *et al.*, 2017).

### Rice

Rice (*Oryza sativa* L.) is another of the world’s leading food crops, with rice, wheat, and maize together accounting for about 51% of human caloric intake (FAO, 2018). In recent years an ever-growing number of studies have focused on the epigenetic changes that are associated with the responses of rice to abiotic stresses. Initial evidence for an association between abiotic stress responses and stress-induced epigenetic variation was provided by Wang *et al.* (2011*a*) who showed that drought-tolerant and drought-susceptible rice cultivars displayed differential site-specific DNA methylation upon drought imposed at the tillering stage, which was manifested in a genotype- and tissue-specific manner. More recent studies employing ‘-omics’ technologies have highlighted the association between altered DNA methylation patterns and differential gene expression at the genome-wide level in three rice cultivars with distinct susceptibilities to increased salt and drought stresses (Garg *et al.*, 2015). Methylation-sensitive amplification polymorphism (MSAP) analysis detected drought-induced genome-wide DNA methylation changes that accounted for about 12% of the total site-specific methylation difference across genotypes, tissues, and developmental stages of which nearly 70% were reversed upon recovery and 29% were maintained (Wang *et al.*, 2011*a*). Overall, drought appeared to induce DNA demethylation events that were more pronounced in the tillering stage and varied among tissues and developmental stages between the two cultivars studied (Wang *et al.*, 2011*a*). In another MSAP study, when drought was imposed at the early reproductive stage (panicle initiation), hypomethylation was found to be more pronounced in a drought-tolerant rice genotype, whereas hypermethylation events were evidenced in a drought-susceptible genotype (Gayacharan and Joel, 2013).

DNA methylation alterations were detected in salt-sensitive and salt-tolerant rice varieties upon exposure to increased salinity (Wang *et al.*, 2011*b*). Subsequent MSAP studies on the association of DNA methylation and rice salt tolerance demonstrated that high salinity conditions induced changes in the DNA methylation pattern within gene bodies in the genomes of contrasting rice genotypes and in certain instances differential DNA methylation was accompanied by altered gene expression in a cultivar-dependent manner (Karan *et al.*, 2012). In a very recent report, DNA immunoprecipitation with the 5-methylcytosine antibody and high throughput sequencing (MeDIP-seq) was utilized to uncover the genome-wide methylation status of a salt-tolerant rice variety under increased salinity (Ferreira *et al.*, 2019). A series of differentially methylated regions (DMRs) were identified between the control and salt-exposed plants and a general tendency was evidenced for demethylation events induced by salt imposition, in agreement with previous reports (Wang *et al.*, 2011*b*; Ferreira *et al.*, 2015). Moreover, DMRs were associated with specific genes and differential DNA methylation within or in the vicinity of the genes with affected transcriptional capacity (Ferreira *et al.*, 2019). These findings revealed new epigenetic factors and target genes that are associated to the response to salt stress in rice and may be further exploited towards enhanced resilience under salt stress conditions.

Extensive information has been reported in the past several years regarding the role of miRNAs in rice response to abiotic stress (Grewal *et al.*, 2018; Jeong and Green, 2013; Shriram *et al.*, 2016; Nadarajah and Kumar, 2019; Patel *et al.*, 2019; Xu *et al.*, 2019). Yang *et al.* (2013) reported that overexpression of two rice miRNAs of the miR319 family, *osa-mir319a* and *osa-mir319b*, led to morphological changes, such as wider leaf blades and increased number of longitudinal small veins, and conferred enhanced tolerance to cold stress in the transgenic rice lines (Yang *et al.*, 2013). Similarly, overexpression of a *miR156* resulted in increased rice tolerance to salt stress and reduced expression of the transcription factor target genes *SQUAMOSA PROMOTER BINDING ROTEIN LIKE-9* (*SPL9*) and *DIHYDROFLAVONOL-4-REDUCTASE* (*DFR*) mostly associated with developmental processes (Cui *et al.*, 2014). It was postulated that *miR159* acts as cellular regulator that directs either developmental or stress responses depending on the need to counteract external stressful conditions. In another study, knock-down of *miR166* led to increased resistance of the transgenic rice lines to drought accompanied by morphological changes associated to the plant’s natural drought responses, such as leaf rolling and decreased xylem diameter (Zhang *et al.*, 2018).

### Legume crops

The three most thoroughly studied legume species are *Glycine max* (soybean), *Medicago truncatula*, and *Lotus japonicus* due to their economic significance or their importance as genomic models (Mochida *et al.*, 2010; Cañas and Beltrán, 2018; Ramesh *et al.*, 2019). Genomic tools have recently been developed for many other highly important legumes, such as *Phaseolus vulgaris* (common bean), *Cicer arietinum* (chickpea), and *Vigna unguiculata* (cowpea) (Timko *et al.*, 2008; Varshney *et al.*, 2013; Lobaton *et al.*, 2018).

However, research into legume responses to abiotic stresses in relation to DNA methylation is very limited. Labra *et al.* (2002) reported hypermethylation of cytosine residues in the DNA of *Pisum sativum* root tips under water deficit conditions compared with well-watered plants. Inversely, heat stress resulted in hypomethylation of cytosine in the DNA of *Glycine max* roots (Hossain *et al.*, 2017). In a more detailed study, Abid *et al.* (2017) compared the amount of methylation in a drought-resistant and a drought-sensitive genotype of *Vicia faba* under water deficit conditions. Demethylation was recorded under drought stress and consequently the tolerant genotype had a higher rate of demethylation compared with the sensitive one. Furthermore, comparing the methylation rate with the related gene expression, they suggested that for drought-responsive genes up-regulated through demethylation, the extent of demethylation was related to drought stress tolerance.

A plethora of drought-responsive miRNAs have been identified in a variety of legume species either by utilizing traditional methods or by high-throughput miRNA deep sequencing (Mantri *et al.*, 2013). Khandal *et al.* (2017) identified 259 miRNAs by deep sequencing that were differentially expressed in root apex of chickpea under drought and salinity stress. Some of these are expressed in the same manner also in *Medicago* and soybean root tips subjected to salt treatments, while others show different expression patterns. Interestingly, many of these hold auxin- and abiotic stress-responsive *cis*-elements in their promoters and thus their regulation is controlled by the accumulation of phytohormones. Hajyzadeh *et al.* (2015) reported that *miR408* transcripts were accumulated under drought stress in *Cicer arietinum*, which is in agreement with similar studies conducted in several other plant species such as *H. vulgare*, *M. truncatula* (Kantar *et al.*, 2010; Trindade *et al.*, 2010), and Arabidopsis (Liu *et al.*, 2008). Barrera-Figueroa *et al.* (2012) identified 24 novel miRNA families in a drought-tolerant and a drought-sensitive genotype of cowpea. Reported for the first time, six of these families of conserved miRNAs occur in other plant species, and 22 miRNA families have also been found in soybean by Kulcheski *et al.* (2011). These researchers also observed differential expression of 11 miRNAs between two soybean genotypes with differential responses to drought stress. Interestingly, the majority of the identified isomiRNAs were up-regulated during drought stress in the sensitive genotypes, and down-regulated in the tolerant one. Moreover, one of these miRNAs, MIR-Seq11, was found to differentially regulate its target peroxidase protein in the two contrasting genotypes by being up-regulated in the sensitive genotype under drought stress and unchanged in the tolerant one. Thus, the stability of MIR-Seq11 expression levels in the tolerant genotype may contribute to the tolerance. Shui *et al.* (2013) observed an increase of *P5CS* transcripts that induced proline accumulation in a drought-tolerant cowpea variety, which co-occurred with a very low expression of *vun-miR5021* and *vun-miR156b-3p*, which are predicted to target the *P5CS* gene. In alfalfa, Arshad *et al.* (2018) found that *miR156* regulates drought responses by targeting *WD40-2* and affecting other physiological traits. Drought stimulated *miR156* expression, which in turn cleaved the *WD40-2* transcript in alfalfa. (Arshad *et al.*, 2018). These two studies highlight the fact that although many miRNAs are conserved across different plant species, their targets may not be. De la Rosa *et al.* (2019) demonstrated a dicistronic arrangement of *miR398a* and *miR2119* in common bean, and their co-repression due to drought stress controls the coordinated up-regulation of the targeted transcripts encoding COPPER ZINC SUPEROXIDE DISMUTASE 1 (CSD1) and ALCOHOL DEHYDROGENASE 1 (ADH1), respectively, which contribute to an adjustment to water deficit conditions. Another shared mechanism between legume species that regulates plant responses to water deficit is *miR1514a*-mediated regulation of a NAC transcription factor transcript through phased siRNA (phasiRNA) production. Results by Sosa-Valencia *et al.* (2017*a*) in *Phaseolus vulgaris* demonstrate that the terminal drought-resistant cultivar PS targets a NAC transcription factor mRNA, through *miR1514a* induction, leading to NAC 700 transcript cleavage and phasiRNA production. Similarly, *miR1514a* modulation of a NAC transcription factor transcript that triggers phasiRNA formation in response to drought has been found in *M. truncatula* and in *G. max* as well (Arikit *et al.*, 2014; Sosa-Valencia *et al.*, 2017*b*). Thus, a better understanding of the functional significance of such conserved drought-responsive miRNAs in legumes will help in identifying universal biomarkers for use in legume breeding (Ramesh *et al.*, 2019).

Besides the conserved drought-responsive miRNA complexes in different legume species, the specific mechanisms that occur in some legume species are of great importance. A very interesting study conducted by Jatan *et al.* (2019) investigated the impact of a root-colonizing PGPR (*Pseudomonas putida* strain MTCC5279 (RA)), with known ability to ameliorate plant growth and development, in controlling the regulation of miRNAs and their target genes and therefore in improving drought tolerance in chickpea (Tiwari *et al.*, 2016). Thus, through high throughput sequencing they identified RA-responsive miRNAs and their target genes (i.e. *miR8175-ERF7*, *miR166-HD-ZIP III*), which can help scientists elucidate the adaptive responses of chickpea plants to drought stress through RA-mediated regulation of expression of miRNAs (Jatan *et al.*, 2019).

### Rapeseed

Rapeseed (*Brassica napus* L.) is the second most important oilseed crop of the world, with total world production of 75 million tonnes in 2018 (FAO, 2018). However, its production is often limited by abiotic stresses such as drought, salinity, and low temperature. Similar to wheat, rapeseed is an allopolyploid, and an interesting crop species for epigenetic analysis since it was obtained from hybridization between *B. rapa* and *B. oleracea* and its genetic diversity is not at a high level due to its short history of domestication and further narrowing of diversity by breeding. As variation on the DNA level is not high, changes on the epigenetics level may be of importance for improving rapeseed and its response to climate change. Most of the work performed on the epigenetics of rapeseed has involved analysing changes in methylation level under stress. Differences in epigenetic methylation were found between distinct genotypes (Gao *et al.*, 2014). DNA methylation and demethylation were proven to affect expression of a large number of genes when exposed to heat. While in a heat-tolerant rapeseed variety more DNA demethylation was observed, the opposite was observed in a heat-sensitive genotype (Gao *et al.*, 2014).

During salt-induced stress, more extensive DNA demethylation was observed in a salt-tolerant rapeseed genotype, while more extensive DNA methylation was observed in a salt-sensitive genotype after exposure to increased salt concentration in the growth medium (Marconi *et al.*, 2013). Interestingly, different effects on the epigenome are caused by different salt concentrations. Low salt concentration was found to lead to promotion of seed germination and seedling growth (Fang *et al.*, 2017). On an epigenomic level, this phenotype was expressed by decrease in DNA methylation and H3K9me2 and enrichment of H3K4me3. Higher salt concentrations of 50 and 100 mM caused a completely opposite effect on both the phenotypic and the epigenetic level. In general, more DNA demethylation in stress-tolerant varieties may be explained by studies that show that crop genotypes that avoid cytosine methylation could be agriculturally superior compared with the ones that are prone to methylation (Gao *et al.*, 2014). Thus, the information on how much a crop genotype is prone to methylation may be of importance for crop improvement for increased abiotic stress resilience.

### Tomato

Tomato (*Solanum lycopersicum* L.) is one of the world’s most cultivated vegetable species. One of the first tomato epimutants characterized was the colourless non-ripening (*Cnr*) mutation, which inhibits ripening of tomato fruits (Nogueira, 2014). The molecular basis of *Cnr* is the hypermethylation of an *SBP3*-like transcription factor gene (Manning *et al.*, 2006). The epimutation is inherited relatively stably across generations, highlighting the developmental relevance of DNA methylation in tomato (Manning *et al.*, 2006).

Dynamic changes in DNA methylation were also demonstrated under drought stress. In particular, *Asr* (absisic acid, stress, ripening) genes have been studied in this respect (Frankel *et al.*, 2006). They encode putative transcription factors that are up-regulated upon abiotic stress exposure. In tomato, Asr1 and Asr2 both increase in expression upon drought stress, and the expression change correlates with demethylation of putative regulatory and transcribed regions (González *et al.*, 2011, 2013). It was recently demonstrated that drought stress leads to the activation of long terminal repeat retrotransposons of the *Rider* family in tomato (Benoit *et al.*, 2019). Epigenetic pathways including RNA-dependent DNA methylation appear to play a role in *Rider* silencing. *Rider* transposons are an important source of phenotypic variation in tomato (Benoit *et al.*, 2019), and it will be interesting to see whether they can be used in future tomato breeding approaches.

Ubiquitous methylation changes during development and abiotic stress exposure in tomato, together with evidence of the functional relevance of methylation changes for at least some genes, led to the suggestion of taking epigenetic variation between different tomato varieties into account for breeding programmes (Zhong *et al.*, 2013). The feasibility of epigenetic breeding was further reinforced by transgenic tomato lines in which the plastid and mitochondrial gene *MutS HOMOLOG1* (*MSH1*) was down-regulated by RNAi (Yang *et al.*, 2015). These transgenic plants displayed a variety of altered phenotypes, including increased heat tolerance. All of the altered traits showed incomplete penetrance. Thus, a variety of plants with different characteristics were generated using the *MSH1* RNAi transgene (Yang *et al.*, 2015). Interestingly, the altered phenotypes were inherited independently of the transgene. The stable inheritance of the traits is probably at least to some extent due to altered DNA-methylation patterns (Yang *et al.*, 2015).

It was also demonstrated that chilling induces methylation changes in tomato fruits (Zhang *et al.*, 2016). Promoter regions of genes implicated in fruit ripening seemed to increase in DNA methylation upon chilling. This increase in methylation was correlated with a decrease in expression of the respective genes. Those expression changes, in turn, have been linked to decreased levels of flavour-associated volatiles (Zhang *et al.*, 2016).

## Future prospects: applications in climate-smart crop breeding

As climate change is expected to increase the prevalence of extreme environmental conditions, improved stress tolerance has become a major breeding target. In field conditions, crops are often simultaneously challenged by different biotic and abiotic stresses. Therefore, understanding of shared mechanisms contributing to one or more simultaneously occurring stresses has also become an important aspect in improvement of crop productivity under foreseeable complex stress situations (Ramu *et al.*, 2016). Understanding epigenetic mechanisms and variation could potentially help plant breeders to generate new, more flexible varieties through the exploitation of the natural phenotypic variation in existing crop plants. In addition, environmental buffering effects of epigenetic mechanisms should be exploited to achieve yield stability in a fast-changing climate.

There is growing evidence that epigenetic mechanisms may have a role in increasing crop resilience to specific stresses and therefore may be an important tool in climate-smart crop breeding. For example, methylation of histone H3 lysine 4 (H3K4) is involved in the persistent expression of high temperature-responsive genes, as well as hyper-induction of such genes during repeated heat stress treatments (Lämke *et al.*, 2016). Target gene repression by small non-coding RNAs was found to be activated during plant drought stress. This mechanism was found to be involved in the drought stress response in barley in which in drought-stressed plants the promoter region of *cytokinin-oxidase 2.1* (*HvCKX 2.1*) had an increased level of DNA methylation. Furthermore, the seeds from the drought-stressed plants showed faster shoot emergence due to an abundance of cytokinin ribosides (Surdonja *et al.*, 2017). In rice, it was found that a high proportion of the drought-induced epimutations (DNA methylation changes) maintained their altered methylation pattern in successive generations exposed to drought from the tillering to the grain filling stage, suggesting the presence of possible epi-marks that are drought inducible and heritable across generations (Zheng *et al.*, 2017). Together with the observation that multigenerational drought exposure improved the adaptability of rice plants to drought conditions, these findings suggested that epigenetic modifications may play important roles in the response to drought and the long-term adaptation of rice, and perhaps other plants, to adverse environmental conditions (Zheng *et al.*, 2017).

### Potential challenges

Use of natural or induced epigenetic variations in creation of climate-smart crops requires that these variations are stable and heritable, two features of major importance for potential transmission to the progeny (Eichten *et al.*, 2014; Iwasaki and Paszkowski, 2014; Vriet *et al.*, 2015). Most of the stress-induced epigenetic modifications return to initial levels when the stress is removed, but some of the modifications might be stable and inherited across mitotic or even meiotic cell divisions (Sudan *et al.*, 2018). There are several reports on epigenetically mediated stress memory that later led to long-term adaptation. For instance, Blodner *et al.* (2007) reported that exposure of Arabidopsis plants to cold stress during flowering and seed development resulted in improved photosynthetic yield recovery in their progeny in response to chilling conditions. Furthermore, Cortijo *et al.* (2014) found that induced methylation variants in Arabidopsis are stable over time scales necessary to breed novel traits, so much so that quantitative trait loci (QTLs) for some phenotypes have been identified using DMRs as genetic markers. However, further study of the factors affecting epiallele stability in crops is needed in order to avoid inducing epialleles that are unlikely to be stable during the breeding process (Hofmeister *et al.*, 2017).

One of the tools to overcome this problem could be development of mathematical models for the increase and identification of heritable epigenetic phenotypes, which could also enhance the efficiency of breeding programmes. Such mathematical models for better understanding of the evolutionary dynamics and responses to ecological adaptations, which combine information on the probability of transmission of ancestral phenotypes, the number of epigenetic reset opportunities between generations, and assumptions on the environmental induction of the epigenetically regulated trait, have recently been proposed (Tal *et al.*, 2010). They should facilitate the identification of the heritable epigenetic variance and transmissibility for future molecular studies such as genome wide association and QTL studies (Hauser *et al.*, 2011). Another approach could be that proposed by Hofmeister *et al.* (2017) who developed an epigenotyping procedure that enabled uncoupling of newly formed epialleles from potential genetic causes and accurate identification of spontaneous epialleles, as well as better understanding of patterns of epiallele inheritance.

One of the major challenges in creating epigenetic populations in crops by using approaches developed in model species is whether mutations in the DNA methylation mechanism are tolerated, since no viable mutants with changed DNA methylation mechanism have been produced in crops (Hu *et al.*, 2014; Li *et al.*, 2014; Kawakatsu and Ecker, 2019), as a consequence of the higher presence and different chromosome location of transposon sequences in crop genomes compared with genome of Arabidopsis, for instance. An alternative strategy could be to use more precise approaches that have been developed in recent years, such as epimutagenesis and targeted epigenome editing, and directly engineer the epigenome, as has been described in Arabidopsis (Johnson *et al.*, 2014; Springer and Schmitz, 2017). The complex nature of most of the crop genomes will require a combination of technical and biological innovation in order to realize the potential of epigenomic variants and use them efficiently in breeding for improved adaptation to abiotic stresses and for other agronomically important traits.

For further integration of epigenetics and epigenomics in crop breeding, more work needs to be done to create new, reliable, and efficient ways to move beyond correlation between epigenetic variation and the desired trait. This will further enable breeders to use targeted, gene-specific modifications to the epigenome that indeed lead to the anticipated responses and desired phenotypes. Apart from positive effects, stress memory could also have a negative impact on crop yield by preventing the plant from growing to its full potential, which also should be taken into account when using epigenetic variation for developing abiotic stress-tolerant crop varieties (Chinnusamy and Zhu, 2009). One possible approach could be the use of models for prediction of the impact of epigenetic variations on plant phenotype and performance, as well as assessment of the added value of epigenetic reprogramming for plant improvement as described by Hu *et al.* (2015) and Colicchio *et al.* (2015).

### Crop improvement strategies

Based on the information described in this review, there are several possibilities that should be taken into consideration to improve crop stress tolerance. The identification of epialleles and epigenetic regulatory systems that have functional impacts on agronomic traits can lead to a range of different approaches for epigenetic breeding of crop plants, such as the use of mutant lines (Yang *et al.*, 2015), recurrent epi-selection (Hauben *et al.*, 2009; Greaves *et al.*, 2014), hybrid mimics (Wang *et al.*, 2015*a*), epigenomic selection (Jonas and de Koning, 2013; Oakey *et al.*, 2016), and epigenome editing (Park *et al.*, 2016; Lämke and Bäurle, 2017), as well as exploitation of stress priming mechanisms to induce a constitutively primed state and increase the crop’s ability to tolerate stress without the undesired reduction of biomass accumulation and yield (Lämke and Bäurle, 2017).

A few approaches have been described for exploitation of epigenetic variation for crop improvement. A breeding strategy was suggested by Raju *et al.* (2018) for exploitation of epigenetic variations for increasing yield and stability in soybean. The authors used the *MSH1* system to induce epigenetic variation, and developed epi-lines by crossing between wild type and *msh1*-acquired soybean memory lines, with a wide variation for multiple yield-related traits in both greenhouse and field trials. Furthermore, obtained epitypes had low epitype–environment interaction, indicating higher yield stability and a lower effect of environmental constraints. The authors provided evidence that novel epigenetic variation induced by *MSH1* suppression, following crossing and F_2_ segregation, can be inherited for at least three generations and bred for crop improvement with a few rounds of selection to enhance and stabilize crop yield. Raju *et al.* (2018) also identified genes involved in various metabolic pathways responsible for enhanced growth behaviour across generations, confirming that *MSH1*-based epigenetic variation could be of use in plant breeding for enhanced yield and yield stability and that environmentally induced epigenetic variation can result in heritable phenotypic plasticity, which may play a major role in adaptation to changes in the environment (Fujimoto *et al.*, 2012; Robertson and Wolf, 2012).

Further evidence that *MSH1*-based epigenetic variation could be of use in plant breeding was provided by Yang *et al.* (2015). The authors have investigated gene silencing of *MSH1* in tomato cv ‘Rutgers’ and its effect on the developmental reprogramming of the plant. Crossing these transgene-null, developmentally altered plants to the isogenic cv ‘Rutgers’ wild type resulted in progeny lines that show enhanced, heritable growth vigour under both greenhouse and field conditions. This boosted vigour was also graft transmissible and could be partially reversed by treatment with the methylation inhibitor 5-azacytidine, implying the involvement of mobile, epigenetic factors and DNA methylation changes in the underlying molecular mechanism.

In another study, Hauben *et al.* (2009) showed that energy use efficiency is a distinct feature of plant vigour and yield in rapeseed and that it possesses an epigenetic component that can be directed by artificial selection. The main difference compared with the selection used in classical breeding schemes is that epigenetic selection is not only done at the population level, but primarily at the plant level and in a recursive manner. The authors generated populations with distinct physiological and agronomic characteristics from an isogenic canola population of which the individual plants and their self-fertilized progeny were recursively selected for respiration intensity. These populations were found to be genetically identical, but epigenetically different. Furthermore, both the DNA methylation patterns and the agronomic and physiological characteristics of the selected lines were heritable, although some subpopulations returned to their original state in the first rounds of selection. Hybrids derived from parental lines selected for high energy use efficiencies had a 5% yield increase on top of heterosis. The results of this study further demonstrate that artificial selection allows an increase in yield potential by selecting populations with particular epigenomic states. Another important outcome of this study is the finding that recurrent selection can be an efficient tool for fixation of epigenetic traits, information of great practical value for the further use of epigenetic variation in crop breeding.

### Future perspectives

Combined with classical genetic studies, newly available sequencing technologies are facilitating the study of epigenetic phenomena at the whole genome level in a way that was unthinkable only a few years ago. The application of epigenome profiling and engineering could generate new avenues for using the full potential of epigenetics in crop improvement. This, along with the new epigenome editing tools, could enable the creation of novel epiallelic variants by alteration of DNA methylation or other chromatin modifications and crop improvement through epigenome engineering (Springer and Schmitz, 2017).

Genome-wide mapping of epigenetic marks and epigenetic target identification are currently two major strategies in many important crops: these will offer breeders new tools to increase and manipulate epigenomic variability for selecting novel climate-smart crop varieties that are more resilient to environmental changes. Genome-wide mapping of epigenetic marks led to the development of epigenomics, an emerging field that is expanding our ability to explain observed phenotypic variation through the identification of multiple cellular products such as RNAs, protein–DNA interactions, chromatin modifications, and chromatin accessibility (Lane *et al.*, 2014). Collecting and normalizing plant epigenomic data for a range of species will facilitate cross-species comparisons, annotation of genomes, and an understanding of the role of epigenomic functions in crop response to stress (Lane *et al.*, 2014).

Different techniques to modify the epigenome globally or at target sites can be used for induction of epigenetic modifications useful for crop improvement. In recent years, the emergence of CRISPR/Cas9 and dCas technology has provided new routes in the epigenetic field. This new tool enables targeted manipulation of epigenetic characters and could be used to specifically modify plant phenotype or to elucidate the relationship between the epigenome and transcriptional control (Hilton *et al.*, 2015; Moradpour and Abdulah, 2020). The recent advances in the development of engineered DNA-binding domains may make locus-specific epigenetic breeding technology even more precise. The engineered DNA-binding domains such as zinc fingers, transcription activator-like effectors (TALEs), and the endonuclease-deficient Cas9 (dCas9) protein can be used in combination with either activator or repressor domains to introduce permissive or repressive chromatin marks at particular loci and broaden our knowledge of how the activation or repression of particular chromatin regions affects the plant phenotype during stress exposure (Bilichak and Kovalchuk, 2016). The knowledge gained could be further used for long-term activation or repression of a chosen gene or pathway for trait improvement purposes in crops, which along with the use of epigenetic tools such as epigenetic QTLs or epigenetic single nucleotide polymorphisms could lead to the development of a new, efficient, and genetically modified-organism-free breeding method (Bilichak and Kovalchuk, 2016).

All these new technological advances have facilitated exploitation of epigenetic variation in crop breeding and acceleration and more efficient creation of climate-smart crop varieties. For optimal use of these new tools in plant breeding, further studies are needed for specific traits and crops in order to gain more knowledge on the association of stress-induced gene expression changes with alterations in DNA methylation and histone modifications, the mode of inheritance of these modifications, and their adaptive value (Chinnusamy and Zhu, 2009).

In the future, breeders will certainly have to pay more attention to crop epialleles and their potential role in adaptation to changes in environment. Furthermore, defining the molecular basis of transgenerational epigenetic inheritance could ultimately lead to development of epialleles designed for specific environmental conditions through targeted epigenetic modifications in genes of interest. However, more work on a greater range of plant species is needed in order to gain a more comprehensive understanding of the mechanisms inducing and stabilizing epigenetic variation in crops. This will require a combined and multidisciplinary effort of researchers involved in different areas of plant science and better integration of epigenomic data obtained in different crops.
